# Analysing Psychosocial Difficulties in Depression: A Content Comparison between Systematic Literature Review and Patient Perspective

**DOI:** 10.1155/2014/319634

**Published:** 2014-06-09

**Authors:** Kaloyan Kamenov, Blanca Mellor-Marsá, Itziar Leal, Jose Luis Ayuso-Mateos, Maria Cabello

**Affiliations:** ^1^Instituto de Salud Carlos III, Centro Investigación Biométrica en Red (CIBER), C/Diego de León 62, 28006 Madrid, Spain; ^2^Departamento de Psiquiatría, Universidad Autónoma de Madrid, C/Arzobispo Morcillo 4, 28029 Madrid, Spain; ^3^Instituto de Investigación de la Princesa (IIS-IP), Hospital Universitario de la Princesa, C/Diego de León 62, 28006 Madrid, Spain

## Abstract

Despite all the knowledge on depression, it is still unclear whether current literature covers all the psychosocial difficulties (PSDs) important for depressed patients. The aim of the present study was to identify the gaps in the recent literature concerning PSDs and their related variables. Psychosocial difficulties were defined according to the World Health Organization International Classification of Functioning, Disability and Health (ICF). A comparative approach between a systematic literature review, a focus group, and individual interviews with depressed patients was used. Literature reported the main psychosocial difficulties almost fully, but not in the same degree of importance as patients' reports. Furthermore, the covered areas were very general and related to symptomatology. Regarding the related variables, literature focused on clinical variables and treatments above all but did not report that many psychosocial difficulties influence other PSDs. This study identified many existing research gaps in recent literature mainly in the area of related variables of PSDs. Future steps in this direction are needed. Moreover, we suggest that clinicians select interventions covering not only symptoms, but also PSDs and their modifiable related variables. Furthermore, identification of interventions for particular psychosocial difficulties and personalisation of therapies according to individuals' PSDs are necessary.

## 1. Introduction

Depression is a major public health issue due to its prevalence, high mortality rates [1], suicide risk [2], and economic impact on the society [3]. It is considered to be the major cause of years lived with disability (YLD) and by 2020 is expected to be among the two main causes of disability adjusted years (DALYs) together with ischemic heart disease [4]. The functional limitations caused by depression are equal to or even sometimes greater than the ones engendered by many other chronic medical conditions [5]. In spite of the great advances achieved in treatment of depression during the last decades, between 20 and 30% of cases are not adequately solved by first agent treatments (also known as treatment-resistant depression in literature) [6].

This evidence suggests that usual management strategies do not address sufficiently relevant areas of depression. One substantial dimension of depression comprises the psychosocial difficulties (PSDs) which people experience. PSDs constitute the impairment on psychological and social daily functioning of individuals, linked with their particular health condition [7]. The broad range of PSDs encompasses not only the personal, but also the economic and social impacts of the disorder. Therefore, it is of extreme importance that these psychosocial difficulties and their related variables are analyzed accordingly. Such information can throw light on patients' real needs, can help clinicians determine the areas in depression that are to be improved and investigated further, may help to prevent the onset of disability, and last, but not least, may guide policy makers to design better health policies.

Previous scientific literature provides different definitions of psychosocial difficulties in depression but has some limitations: the methods either focus only on specific areas or consider the PSDs as a result of depression. Recent studies [7–9], however, provide a new definition of PSDs, based on WHO's International Classification of Functioning, Disability and Health (ICF) [10]. This new approach covers the whole course, determinants and onset of psychosocial problems, and embodies an innovative holistic model of health. More specifically, it states that PSDs are “…impairments of mental functions, activity limitations and participation restrictions that include both the individual's mental capacities and his or her social interactions (such as in work, family life and leisure activities)” [7]. Moreover, impairments of body functions under central nervous system control such as pain and sexual interest problems are also included as PSDs. A detailed description of the utility of the ICF classification for depression can be found elsewhere [11].

Once this comprehensive definition of psychosocial difficulties has been extracted from the literature, it is very important to incorporate the patient perspective in clinical practice and research. The majority of studies on this topic are quantitative; however, the literature that uses qualitative methodologies—which could provide deeper, richer, and more elaborated data, exploratory analyses on patients' needs and standpoints, and thus more objective results—is sparse. Furthermore, this kind of studies can provide a general view if particular psychosocial difficulties, named by patients as important, are missing in the literature.

There are few existing studies using qualitative research to identify a full list of PSDs which depressed people experience. Yet, none of them has applied a comprehensive approach in order to encompass all the psychosocial difficulties from different perspectives. An example is a study by Lasch et al. [12], which aimed to develop a specific questionnaire to assess symptoms for adult major depressive patients and to track their functional status. The authors have conducted ten focus groups and individual cognitive interviews and identified several general domains containing different concepts. However, this study does not combine the patient perspective with information from the literature.

An article by Brütt et al. [13] based itself on a three-dimensional design—systematic review of literature, patient focus groups, and an expert panel aimed to identify a core set of activities and participation for individuals with mental disorders. Moreover, this research paper relied on the International Classification of Functioning, Disability and Health (ICF) as a conceptual framework for describing functional impairments in patients. Despite the comprehensive approach, the study has focused only on ICF categories of the component activities and participation and has not taken into account the other elements of the framework. Furthermore, it has not included information regarding related variables of psychosocial difficulties in depression.

To date, no study has analyzed the full set of PSDs and their related variables in depression by including both literature and patient perspective. The aim of the present study is to tackle this research gap and discover whether the recent scientific literature actually reports the PSDs and their related variables that are important for depressed patients. Our objective is to obtain information about whether the latest literature extensively covers the issues that are pointed out as problematic by depressed individuals, or if there are particular areas, which should receive more attention. Moreover, a potential identification of these missing previously-ignored questions in literature will enhance the quality of future research and enable new strategies for treatment and rehabilitation in depression.

## 2. Materials and Methods

In order to compare whether the psychosocial difficulties experienced by depressed patients are actually reported by recent literature in depression, we included a step by step methodology.

### 2.1. Extraction of the Information

We gathered information from three different studies.

#### 2.1.1. Systematic Literature Review

First, a systematic review to directly collect information reported in recent literature, consulting the MEDLINE and PsycINFO databases, was conducted. Search terms were adapted to each database combining the MeSH headings of “Depression,” “Depressive Disorder,” and “Depressive Disorder, Major” with “depress*” (title) in MEDLINE database and (DE=) “Major Depression,” “Recurrent Depression,” and “Depressive disorder” in PsycINFO. For psychosocial difficulties the following keywords were used: “psychosocial*,” exp Quality of life/, exp Personal satisfaction/exp Human activities/exp social support/disabilit*, homelessness, environmental factor*, exp Interpersonal relations/, exp Quality of life/, exp personal satisfaction/, exp human activities/, exp paternalism/, prejudice/, psychosocial deprivation/, social values/, exp Social Problems/, Social Adjustment/, social isolation/stereotyping/, exp Social environment/, exp emotions/, exp family/, exp socioeconomic factors/exp life style/exp Disability evaluation/, exp Communication Barriers/, “Adaptation”, exp Psychological/, exp Aggression/, exp Psychological stress/, exp community (no microbial community)/, Sexual* or intimacy. Inclusion criteria were articles reporting information on psychosocial difficulties in people with a diagnosis of major or minor depression according to DSM-III-TR, DSM-IV, or DSM-IV-TR [14–16], or a depressive episode or depressive disorders according to ICD-10 criteria [17]. Qualitative articles and longitudinal observational and interventional studies were also considered. Additional criteria required the studies to be published in English between 2005 and 2010. Full results of the literature review can be seen elsewhere [7]. Furthermore, studies were also excluded if they were cross-sectional and psychometric or if they had not included a standardized diagnosis of depression. Articles reporting patients with bipolar depression, dysthymia, or postpartum depression were also excluded.

Once all the included studies were selected, we extracted information about the sample characteristics and then collected the variables, the tools, the psychosocial difficulties and their related variables. Related variables in literature refer to determinants of PSDs since only longitudinal studies were included. Determinants in literature were those variables that were longitudinal predictors for incidence or changing of psychosocial difficulties, so consequently a causal relationship with the PSDs can be hypothesized. Extraction of the information was double checked by two independent reviewers in 20% of the articles (MC and BM) (Kappa = 0.80). Kappa's coefficient was calculated according to Fleiss and Cohen rules [20].

#### 2.1.2. Focus Group

On the other hand, the collection of patients' perspectives was performed including information from two studies. The first one consisted of one focus group composed of seven depressed patients. We based our sample size decisions on literature recommendations, “The ideal size of a focus group for most noncommercial topics is five to eight participants” [21] and the concern that a larger group could make participants reserved or there would not be enough time to hear everyone's contribution. Inclusion criteria for participating in the session were patients older than 18 years, with a diagnosis of a depressive episode or a depressive disorder according to ICD-10 during the last year. Eight patients from the* Hospital Universitario de la Princesa* in Madrid were invited to participate. Selection was done by their main mental health care provider (psychiatrist) taking into consideration the maximum variability of sampling in gender, work status, and clinical status (totally remitted, partially remitted, and nonremitted), consulting the patients' clinical records. Eight patients were invited to participate and one of them did not consent to participate reporting having no time for this. Participation in this study was not mandatory and only patients with motivation to participate were included in the final sample; therefore a selection bias could have affected our results. Participation was formally agreed after signing a consent informed form. One moderator and one assistant (MC and IL), who had been previously trained, encouraged all the members to participate during the session.

Four open questions were posed: (1) which psychosocial difficulties are usually experienced due to participant's depression; (2) which ones are more relevant (ranking the five most important ones for each participant); (3) how these difficulties changed overtime; and (4) which events are responsible for the onset or change of these psychosocial difficulties overtime. All the dialogues were digitally recorded and subsequently transcribed.

#### 2.1.3. Individual Interviews

Finally, in order to gather other difficulties that are not usually reported during focus group sessions because of potential unwillingness of self-disclosure [22], we included data from 80 individual interviews. The preselection of participants was done by their health care providers (psychiatrist and GPs) according to the study inclusion and exclusion criteria. All the patients that fulfilled the inclusion criteria were invited to participate and providedbasic information (name and telephone) to researchers to make an appointment. Only four patients did not agree to participate. From these, two justifiedtheir denial with the argument* “I have no time to participate,*” one had no interest in research studies, and one showed no interest in this particular study. During the initial interview researchers verified the fulfillment of the inclusion criteria. At that point five patients that were derived from the primary care center were excluded because they met diagnostic criteria for other different disorders: bipolar disorders (2), generalized anxiety disorder (1), substance use disorder (1), and complicated grief (*n* = 1) and thus satisfied one of the exclusion criteria.

Patients were singly asked, among other questions, which were the five most disabling psychosocial difficulties that they experienced due to depression and which were the variables that were responsible for the onset or the change of these problems. A causal relationship with the PSDs and extraction of determinants, unlike in the literature, cannot be established, because of the nature of the focus group and the individual interviews; therefore we will refer to these variables of onset or change of PSDs according to the patient perspective as related variables.

Trained interviewers (MC, BM) conducted all the individual interviews. Inclusion criteria for individual interviews were patients older than 18 years with a diagnosis of depressive episode or major depression criteria according to ICD-10. All patients were collected in “*Santa Hortensia*” Primary Care center of Madrid or in the outpatient psychiatric service at the Hospital* Universitario de la Princesa* in Madrid. In both study sites patients were chosen according to their availability and were invited to participate by their primary care doctors or psychiatrists. All participants signed an informed consent form. Both studies were independently reviewed and approved by the Hospital Ethics Committee for Clinical Research.

### 2.2. Agreement on the Terminology to Describe PSDs and Their Related Variables

After gathering information from the three different studies, we needed to establish a common language necessary to directly compare outcomes. For that purpose a list of common categories for classifying psychosocial difficulties was agreed on during one research group meeting. Participants were researchers who had been involved in data collection for different health conditions. Researchers involved in the data collection of depression (IL, BM, and MC) also participated. All of them were requested to share with the group the PSDs that they had identified in the different studies and the terminology they had used to name them. After each naming, the working group was asked whether they agreed with the terminology proposed. After a brief discussion, stating pros and cons for the proposal, an agreed-on term was decided and documented for each PSD. The same procedure was followed to extract the names of related variables in subsequent working group sessions with the same participants.

### 2.3. Linking Process of Concepts

After obtaining the list of common categories, we associated the different concepts extracted in the studies to the agreed category list. For* personal factors* we used the definition given by the WHO's International Classification of Functioning, Disability and Health: “Personal factorsare the particular background of an individual's life and living, and comprise features of the individual that are not part of a health condition or health states. These factors may include gender, race, age, other health conditions, fitness, lifestyle, habits, upbringing, coping styles, social background, education, profession, past and current experience (past life events and concurrent events), overall behaviour pattern and character style, individual psychological assets and other characteristics, all or any of which may play a role in disability at any level. Personal factors are not classified in ICF.” [10]. The liking process was made by two independent researchers according to ICF linking rules [23]. BM and MC participated in the linking process for the focus group and literature review information (Kappa = 0.92 and 0.88, resp.,) and KK and MC performed it for individual interviews (Kappa = 0.85). Disagreements on categories were solved consulting a third expert opinion.

### 2.4. Analysis of Data

Focusing on the information extracted from the literature, a frequency analysis was performed regarding how many different studies particularly reported the psychosocial difficulties or related variables. In the case of individual interviews, the number of times that the different participants reported the psychosocial difficulties and their related variables was calculated. Finally, for the focus group, digital recordings were analyzed in order to extract the number of times in which psychosocial difficulties and related variables had been a topic during the session (i.e., number of times that these issues had been repeated by a different participant). These analyses were performed with the software for qualitative research NVIVO.

## 3. Results

### 3.1. Characteristics of the Participants in the Focus Group and the Individual Interviews

Tables 1 and 2 show the characteristics of the participants in the focus group and the individual interviews.

### 3.2. Does Recent Literature Report the Main PSDs?

Comparison between the literature review and the studies reporting patient's opinion showed that literature does cover almost fully the main psychosocial difficulties directly addressed by depressed individuals. There were relatively few PSDs, not covered by the recent scientific literature.  Table 3 summarizes these outcomes.

*Emotional functions* [24–26],* energy and drive functioning *[27–29],* cognitive functions* [30–32],* employment *[33–35], and* relationship with the others* [36–38] were the most common psychosocial problems emerging both in the literature and the patients' answers. The frequency of the appearance of PSDs was also comparatively identical.

However, some main PSDs in the patient reports were not covered enough by the literature.* Carrying out daily routine* was pointed out by ten patients in the individual interviews (making it the fifth most important PSD), but it has been investigated less than three times in the revised literature.

*Communication with others* was among the most important psychosocial problems, according to the participants in the focus group, but our systematic review did not find it mentioned anywhere in the literature. Moreover,* weight maintenance functions* and* doing housework *were highlighted by the patients in the individual interviews, but literature omitted them as significant and important psychosocial difficulties in depression. In addition, there were some main categories, which did not meet the same ranking of significance when the different sources of information were compared.* Pain* [39–41] and* sleep *[42, 43], for example, were among the most important categories according to the literature, but in the focus group and individual interviews they were not emphasized notably by patients.

### 3.3. Specific PSDs

Regardless of these few above mentioned PSDs, literature in general addresses substantially the main psychosocial difficulties in depression. Therefore, we conducted an elaborate second level analysis to investigate whether the specific psychosocial difficulties (components within the main PSD categories), reported in the focus group and individual interviews, were also identified in the literature. Once more, the results demonstrated that the literature, with some exceptions, almost fully covers the range of specific psychosocial difficulties, experienced by depressed patients.

However, as can be seen in Table 4, some specific PSDs were reported with different degrees of importance when the three sources of information were compared.* Loneliness* and* distress* (part of* emotional functions*) were among the most important and commonly mentioned difficulties by the patients, whereas these specific PSDs were reported less than three times in the literature. The same is valid for* attention* and* memory* (part of* cognitive functions*),* efficiency (employment*), and* intimate relationships (relationships with others*).

### 3.4. Does Recent Literature Report the Related Variables of PSDs?

Additionally, we investigated whether literature sufficiently reports the most important related variables of change and onset for psychosocial problems in depression that are addressed by patients. All related variables can be seen in Table 5. Literature reported only few related variables of onset of PSDs. In fact, scientific literature reported clinical variables and treatments above all. In contrast, the patients' perspective, extracted by the focus group and individual interviews we conducted, focused on how particular psychosocial difficulties lead to other PSDs. For instance,* treatment* [44–46] was a related variable of change in several studies as reported in literature, but participants in the focus group identified it also as a related variable of onset for specific PSDs. The same was valid for the role of the emotions in change of PSDs, which was frequently indicated by literature, whereas patients reported these* emotional functions* as a cause of psychosocial difficulties as well. Specifically, results showed that the literature did not analyse sufficiently* cognitive functions*,* relationships with others, energy and drive, *and* employment problems* as causes of PSDs.

## 4. Discussion

The current study aimed to analyse whether the recent scientific literature reports those psychosocial difficulties and their related variables that are important for depressed patients. Our findings indicated that the literature does report almost fully the main psychosocial difficulties, experienced by patients with depression, but the degree of importance of each PSD depended on the source of information. The same refers to the specific PSDs, extracted by a second level analysis. Contrary to the main PSDs reported, however, only a few related variables of onset of PSDs were currently reported in literature. In addition, literature was specifically focused on clinical variables as related to the PSDs, whereas it ignored that some PSDs can also become related variables for other PSDs.

To our knowledge, this is the first study to throw light on whether the latest scientific literature actually reflects the issues that are indicated as problematic by depressed patients. However, all the subsequent comments on the literature have to be considered with respect to the only 103 existing studies within our period of search that covered the whole spectrum of PSDs in depression and the fact that their average quality rate is not high [7]. In this sense, this amount of articles seems insufficient, since the literature review aimed to identify a list of studies examining a wide range of psychosocial problems in depression. It is not in the scope of the current paper to elaborate on this deficit, but, given the present results, it is important to mention that besides the existing information gaps in the specialized publications, the quantity of adequate studies addressing the most common difficulties and related variables represents an additional limitation in the literature.

With regard to the most common psychosocial difficulties, the literature and the patients' outcomes matched almost entirely*. Emotional functions *stood out as an evident problem for depressed patients, as part of the outcomes of 62 research studies and being mentioned more than 100 times during the focus group and individual interviews. A review by Brockow et al. [47], based on the ICF classification, confirms the notion that the emotional functioning is among the most affected areas of depression. A second level analysis, conducted to examine the specific segments of the main categories, however, showed some discrepancies between the literature and the patients' perspective. Incongruence regarding* depressive mood and symptoms* [48–50] (being highlighted in literature whereas downplayed by patients) or feelings of* loneliness* and* distress* (emphasized notably by depressed patients but narrowly explored in the literature) can be explained through the literature tendency to focus mainly on those PSDs which belong to the symptomatology spectrum. In addition, according to our results, the literature usually reported major PSD concepts. However, other more specific and smaller categories, also highly affecting the emotional functioning of individuals—like* loneliness* and* distress*—were neglected.

The same applies to other groups like* cognitive functions*, where research studies have focused on capital subcategories of the main PSDs, such as* cognitive functions in general, thought functions, employment in general, *or* relationships with the others in general*, but have not elaborated on smaller features. According to the present results, patients emphasize the importance of the specific problem they experience, even if it is very distinctive and differential. Therefore, the literature should include adequate instruments to address these particular psychosocial issues and encompass the most delicate and uncovered features of the psychosocial functioning of individuals with depression.

Furthermore, another interesting discrepancy between research and patient perspective regarding the consideration of* pain* and* sleep* as important PSDscan be noticed. Both problems have been frequently emphasized by different research studies as essential for depression, while only few participants in the focus group and individual interviews mentioned them as important. One possible explanation is the hiatus in people's perceptions between depression and physical symptoms. Although somatic symptoms are a common feature of depression [51], a substantial percentage of patients, diagnosed with depression, understand both as separate entities. Moreover, they frequently indicate only physical symptoms as the reason for seeking medical assistance [52]. Therefore, many depressed outpatients in the present studies might have omitted some physical complaints, disregarding any association between them and their depressive condition. Another reason could be the nature of the commonly used outcome instruments in depression studies. Most of them, such as the Hamilton Rating Scale for Depression (HRSD) [53], the Short Form Health Survey (SF-36) [54], or the WHOQOL-100 [55], include physical symptom items. Therefore, outcomes such as pain and sleep disturbances are often included in scientific studies. Further qualitative studies should confirm whether or not these PSDs are really important for depressed patients, because in that case literature might be overestimating their relevance in depression.

Regarding the related variables of PSDs, the results of our analysis revealed interesting and alarming gaps in the recent literature. Essentially, only few related variables of onset of PSDs were reported in research.* Emotional functions*, for example, being the leading related variable of onset for participants in the focus group, have been considered only as a determinant of change in literature. The same applies for* treatment*. Depressed patients very often stated that the cause of their PSDs was the type of treatment they received. As can be extracted from literature, however,* treatment* is only able to change the course of the disease (generally as a facilitator). Literature should therefore consider the positive and negative consequences of treatments on patient's PSDs and not only report the positive impact on them. This type of information would be useful to help clinicians decide among the wide range of interventions available.

On the other hand, current scientific literature concentrates on clinical variables and treatments as the only PSDs' related variables, whereas patients additionally highlight the relationships between different PSDs. Specifically, if the patient perspective is considered, literature on* cognitive functions*,* relationships with others, energy and drive, *and* employment problems* as related variables of PSDs would have to be described as insufficient. These components are fundamental to the understanding of the psychosocial functioning of depressed patients. Hence, if the aim is to reach a general development in this direction, future research needs to focus on these gaps.

Overall, these findings reveal the fact that there is a discrepancy between the patient's and the health science researchers' perspective when analysing not only the biological and psychological factors of depression, but also the socioeconomic and environmental variables. The subject's view of his health and functioning is not usually taken into account when designing research studies in depression. In this sense, future studies may address this wider view in a more accurate way by taking into account additional sources that are primarily focused on the subject and its sociological environment. From a sociological point of view it could be claimed that the mainstream perspective from which most institutions and professionals develop their work is not reflecting the quotidian environmental stressors that interfere with an adaptive and healthy development of everyday life in people with depression. It could be hypothesized that this is a result of the current and most common definition of depression through a list of symptoms facilitated by established diagnostic manuals: the DSM and ICD. They are based on passive categorical labelling (mainly by health care professionals), which list a number of prototype behaviours that could occur if certain psychological problems occur. This conceptualization within the medical model does not take into account the life circumstances and biographical context, which the individual interacts with, and thus the psychological sense that provides the key to understanding how this problem has been generated and why it remains is disregarded.

The ICF model has been created by WHO as a complementary tool to the diagnostic systems (DSM and ICD) to describe the day-to-day functioning of people. Within the ICF model, psychosocial difficulties are seen as a continuum and as a result of the complex interaction of environmental variables, mental functions, personal variables, activities and participation, and health status. Another option for personalization of medicine and treatment in real clinical settings might be the inclusion of evaluations of daily functional problems experienced by patients. Therefore we suggest customization of healthcare—with therapeutic decisions being tailored to the individual patient. The diagnostic testing has to be adjusted accordingly for selecting appropriate therapies. Eventually, engagement of patients in identifying specific personal problems (e.g., dysfunctional patterns of emotional, cognitive, and behavioural reactivity to natural environments in daily life) could provide more personalized information and change the pattern of diagnosing. For example, ecological momentary assessments of patients via different technologies, eventual self-monitoring, or even more collaborative interactions with therapists and professional carers would enhance research in this direction and give us a new insight. Recent literature based on ecological momentary assessments (EMA) gives us promising results in this line. Different studies [56, 57] have found the experience of positive affect to be prominent in resilience against depression and to predict recovery [58]. Depression has been found to influence work performance with significant decrease in task focus and productivity [59]. Evidence shows that patients with major depressive disorder experience fewer positive events and perceive them as more stressful [60]. Among adolescents, depressive symptoms have been found related to less effective emotion regulation [61]. The EMA have been implemented in research not only on depression, but also on schizophrenia, anxiety, ADHD, bipolar disorder, and so forth.

Ecological momentary assessments have been initially used to identify moment-to-moment patterns and mechanisms of psychopathology [62], but with the development of technologies and especially web based applications, real-life data become available to patients and clinicians, making the transformation of implicit real-life patterns into explicit ones possible, thus improving personalized mental health care [63]. The method has been recently successfully implemented in studies, aiming to explore whether self-monitoring can also be used as an intervention to increase patients' insights in personalized patterns of positive affect [64]. The EMA approach is just one promising way of overcoming the discrepancy between the patient's and the health science researchers' perspective when analysing the variety of factors for depression. Its aim is not to replace the traditional face-to-face contact with practitioners but to allow patients to take an active role in their recovery process and to personalize the treatment process.

Moreover, the patient perspective on PSDs and their related variables has also an impact on the clinical arena. Results of this study highlight the need for change and adjustment of future strategies for treatment and rehabilitation in depression. The process could be more productive, when clinicians select interventions covering PSDs and their modifiable determinants or related variables, and not only symptoms. Solving some activity problems, for example, can help in improving other areas as well. Furthermore, personalization of therapies according to individuals' PSDs will enhance the treatment process. In general, identification of specific interventions for particular psychosocial difficulties is necessary and future research should address this issue.

### 4.1. Limitations

The findings of this study, however, have to be interpreted assuming its limitations. First, only one focus group of seven patients was performed and saturation point—considering the quantity of information regarding the analysed PSDs—was not reached; therefore the focus group can be considered by some as nonrepresentative. As a partial solution we included a heterogeneous group through maximum sampling variation regarding sex, working status, and clinical status. The age range, however, was between 44 and 55 years, which might have caused a potential bias in the identification of PSDs. This is an important limitation, since some psychosocial difficulties, usually experienced in particular ages, like physical pain in older adults, or problems with intimate relationships or loss of life goals in younger adults, might have been omitted by the nonpresence of representatives of these age groups in our focus group. During the selection process, the psychiatrists who were responsible for the recruitment of participants took into consideration the maximum variability of sampling, but the time frame and the profile of patients in the catchment area of the Hospital* Universitario de la Princesa* in general did not allow us to have a more representative sample in terms of age. However, we decided to proceed with the focus group, because, in fact, a more homogeneous sample of participants is often preferable in terms of age, since it might increase the group comfort level when discussing sensitive topics. Therefore we could assess a wide variety of PSDs including sexual problems, relationships with the therapist, and somatic problems. Finally, in order to achieve more reliable results, we conducted individual interviews with 80 patients ranging substantially in terms of age. Thereat, in spite of this limitation, we found a high variability of PSDs and the valuable finding about the discrepancies between literature and patient reports. Further studies reaching saturation points should be done to test if there are other underexplored PSDs.

Second, a limited period of time was included for the literature search (studies between 2005 and 2010). The search was performed over papers published within the cited period of time because of temporal limitations and because we were interested in the recent literature outcomes rather than general literature. However, we made replication of the original search from 2011 to September 2013 in order to check if any new PSDs will occur in comparison with the initial search and the results did not identify new PSDs. The results of the review have to be read in light of the limitations due to the type of databases consulted. This review study was a component of a larger project that gathered psychosocial factors from several mental health and neurological conditions where only Medline and PsycINFO were included. As a consequence, an amount of studies might have not been identified. As a limitation concerning the reliability of the extraction process we have to indicate that only 20% of articles were independently double checked. Finally, by limiting our search to English literature, we might have omitted relevant papers in other languages.

## 5. Conclusions

The present study is, to our knowledge, the first to analyse the full set of psychosocial difficulties and their related variables in depression through the literature and the patient perspective. We made an elaborate comparison between both sources of information in order to verify whether recent research literature reported all the PSDs and related variables that are important for depressed patients and to identify the existing gaps in this area. Regarding the research on depression, the results obtained within our study show the existence of many literature gaps and encourage future studies to focus on them more in depth. Concerning the clinical implications, this study emphasizes the need for change and adjustment of future strategies for treatment and rehabilitation in depression. We suggest that clinicians should select interventions that cover PSDs and their modifiable related variables and not only improve symptoms. Furthermore, eventual identification and personalization of therapies according to individuals' PSDs would potentially enhance the rehabilitation process.

## Figures and Tables

**Table 1:** Characteristics of the participants in the focus group.

Case	Gender	Age	Work status	Comorbidity	Mental health status
1	Female	51	Retired	Fibromyalgia	Partial remission
2	Male	44	Self-employed	No	Total remission
3	Male	50	Employed	Hepatitis C	Partial remission
4	Female	50	Disabled	Cancer	Depression
5	Male	49	Unemployed	No	Partial remission
6	Female	46	Disabled	Arthrosis	Total remission
7	Male	55	Retired	No	Depression

**Table 2:** Characteristics of the participants in the individual interviews.

Variables	Setting	
	Specialized care	Primary care
	(*n* = 61, 75.3%)	(*n* = 20, 24.7%)
Age	(*n* = 61)	(*n* = 20)
18–34	4 (6.6%)	4 (20.0%)
35–49	14 (23.0%)	6 (30.0%)
50–64	29 (47.0%)	5 (25.0%)
65+	14 (23.0%)	5 (25.0%)
Gender		
Female	50 (82.0%)	17 (85.0%)
Level of education		
Less than primary school	11 (18.0%)	3 (15.0%)
Primary school completed	9 (14.8%)	4 (20.0%)
Secondary school completed	5 (8.2%)	4 (20.0%)
High School	10 (16.4%)	4 (20.0%)
University	20 (32.8%)	2 (10.0%)
Postgraduate studies completed	6 (9.8%)	3 (15.0%)
Work situation		
Working	14 (23.0%)	8 (40%)
Working (sick leave)	9 (14.7%)	1 (5.0%)
Unemployed	18 (29.5%)	4 (20.0%)
Homemaker	8 (13.1%)	4 (20.0%)
Student	1 (1.6%)	—
Retired	7 (11.5%)	1 (5.0%)
Disability pension	3 (4.9%)	—
Others	1 (1.6%)	2 (10.0%)
Number of previous depressive episodes	(*n* = 48)	(*n* = 80)
0 (first episode)	7 (11.5%)	3 (15.0%)
1-2	26 (41.7%)	11 (55.0%)
+2	15 (24.5%)	2 (10.0%)
Self-administered Comorbidity Questionnaire (SCQ) [18], MD (SD)	12.8 (5.1)	11.8 (5.1)
Hamilton Depression Rating Scale-17 (HDRS)*	(*n* = 59)	(*n* = 20)
Mild	4 (6.6%)	11 (55.0%)
Moderate	13 (21.3%)	8 (40.0%)
Severe	42 (68.9%)	1 (5.0%)
Suicide attempts	(*n* = 57)	(*n* = 19)
No	48 (78.7%)	15 (75.0%)
Yes	9 (14.8%)	4 (20.0%)

*Cutoff points based on [19].

**Table 3:** Ranking of the main psychosocial difficulties according to literature and patient reports.

Literature review (number of studies) [ICF code]	Focus group (number of times the PSDs were a topic) [ICF code]	Individual interviews (number of people) [ICF code]
(1) Emotional functions (62) [b152] (2) Pain (20) [b280–b289] (3) Energy and drive (18) [b130, b640] (4) Cognitive functions (17) [b140–b189, b117] (5) Employment (13) [d845–d859] (6) Relationship with others (13) [d7] (7) Self-care (12) [d5] (8) Sleep (11) [b134] (9) Temperament and personality functions (10) [b126] (10) Perception and experience of social support (7) [pf] (11) Participation in social activities (6) [d920] (12) Self-perception (6) [pf] (13) Psychopathological symptoms (5) [b160, b147, b152, b130] (14) Mobility (5) [d4] (15) Locus of control (4) [pf] (16) Psychomotor functions (4) [b147] (17) Driving (3) [d475] (18) Perception and experience of stigma (3) [pf] (19) Self-efficacy (3) [pf] (20) Coping strategies (3^∗^) [pf]	(1) Emotional functions (49) [b152] (2) Cognitive functions (11) [b140–b189, b117] (3) Employment (11) [d845–d859] (4) Perception and experience of social support (9) [pf] (5) Self-perception (9) [pf] (6) Energy and drive (8) [b130, b640] (7) Communication with others (7) [d350] (8) Participating in social activities (6) [d920] (9) Relationship with others (6) [d7] (10) Coping strategies (5) [pf] (11) Psychopathological symptoms (4) [b160, b147, b152, b130] (12) Self-care (4) [d5] (13) Carrying out daily activities (3) [d230] (14) Perception and experience of stigma (3) [pf] (15) Sleep (3) [b134]	(1) Emotional functions (52) [b152] (2) Energy and drive (31) [b130, b640] (3) Relationship with others (24) [d7] (4) Cognitive functions (23) [b140–b189, b117] (5) Carrying out daily routine (10) [d230] (6) Self-perception (8) [pf] (7) Pain (7) [b280–b289] (8) Employment (6) [d845–d859] (9) Participating in social activities (5) [d920] (10) Sleep (5) [b134] (11) Weight maintenance functions (5) [b530] (12) Mobility (5) [d4] (13) Doing housework (3) [d640, d630]

*Only PSDs addressed more than three times in the literature or mentioned more than three times by the participants in the focus group and the individual interviews are shown here.

**Table 4:** Ranking of the specific psychosocial difficulties according to literature and patient reports.

Main PSD categories	Literature review (number of studies)	Focus group (number of times the PSDs were a topic)	Individual interviews (number of persons mentioned the PSD)
Emotional functions	Depressive mood and symptoms (54) Anxiety (8) In general (5) Emotional regulation (3^∗^)	In general (20) Anxiety (10) Depressive mood and symptoms (7) Loneliness (6) Distress (4)	Depressed mood (14) Anxiety (14) Emotional regulation (9) Loneliness (8) In general (5)

Cognitive functions	In general (5) Thought functions (5) Executive functions (3)	Thought functions (6) Attention (3)	Attention (10) Memory (7)

Employment	In general (10)	In general (11)	Efficiency (5) In general (3)

Energy and Drive	Fatigue (8) Vitality (7) Libido (3) Motivation (3)	In general (8)	Motivation (3) In general (10) Fatigue (9) Family (10)

Relationship with others	In general (6) Family (5)	In general (6)	Intimate relationships (9) In general (4)

*Only PSDs addressed more than three times in the literature or mentioned more than three times by the participants in the focus group and the individual interviews are shown here.

**Table 5:** Related variables of psychosocial difficulties in literature and patient reports.

Literature review (type of determinant and number of studies)	Focus group (number of times PSD was a topic)	Individual interviews (number of people mentioned with PSD)
Patient treatment (DC*) (26) Health condition: symptoms (DC) (16) Health condition: severity (DC) (8) Temperament and personality (DC) (6) Comorbidity (DC) (5) Perception and experience of social support (DC) (5) Patient treatment ∗ treatment duration (DC) (4) Emotional functions (DC) (3) Overall score: ADL (DC) (3) Health condition: duration of episode (DC) (3) Stressful life events (DC) (3) Stigma (DO) (3) Self-perception (DO) (3^∗∗^)	Emotional functions (RVO) (18) Stressful life events (RVO) (7) Employment (RVO) (7) Cognitive functions (RVO) (7) Perception and experience of social support (RVO) (6) Energy and drive (RVO) (5) Temperament and personality (RVO) (4) Self-perception (RVO) (4) Comorbidity (RVO) (4) Patient treatment (RVC) (4) Self-perception (RVO) (4) Patient treatment (RVO) (3)	Relationship with others (RVC) (29) Patient treatment (RVC) (23) Emotional functions (RVC) (19) Stressful life events (RVC) (7) Health condition: symptoms (RVC) (7) Attitudes (RVC) (7) Comorbidity (RVC) (6) Energy and drive (RVC) (6) Self-perception (RVC) (5) Coping strategies (RVC) (4) Participation in social activities (RVC) (4) Ageing (RVC) (3) Cognitive functions (RVC) (3) Employment (RVC) (3)


*DC, determinant of change; DO, determinant of onset; RVC, related variable of change; RVO, related variable of onset.

**Only PSDs addressed more than three times in the literature or mentioned more than three times by the participants in the focus group and the individual interviews are shown here.
